# Interlaboratory comparison of a multiplex immunoassay that measures human serum IgG antibodies against six-group B streptococcus polysaccharides

**DOI:** 10.1080/21645515.2024.2330138

**Published:** 2024-04-12

**Authors:** Kirsty Le Doare, Michelle A. Gaylord, Annaliesa S. Anderson, Nick Andrews, Carol J. Baker, Shanna Bolcen, Arif Felek, Peter C. Giardina, Christopher D. Grube, Tom Hall, Bassam Hallis, Alane Izu, Shabir A. Madhi, Pete Maniatis, Mary Matheson, Fatme Mawas, Andrew McKeen, Julia Rhodes, Bailey Alston, Palak Patel, Stephanie Schrag, Raphael Simon, Charles Y. Tan, Stephen Taylor, Gaurav Kwatra, Andrew Gorringe

**Affiliations:** a Centre for Neonatal and Paediatric Infection, Institute for Infection and Immunity, St George’s, University of London, London, UK; b Makerere University Johns Hopkins University, Kampala, Uganda; c UK Health Security Agency, Porton Down, UK; d Pfizer Vaccine Research & Development, Pearl River, NY, USA; e Immunisation and Vaccine Preventable Diseases Division, United Kingdom Health Security Agency (UKHSA), London, UK; f Department of Pediatrics, Division of Infectious Disease, McGovern Medical School and UT Health, Houston, TX, USA; g The Centers for Disease Control and Prevention (CDC), Atlanta, GA, USA; h Vaccine Division, Scientific Research & Innovation Group, MHRA, Potters Bar, UK; i South African Medical Research Council: Vaccines and Infectious Diseases Analytics Research Unit, Faculty of Health Sciences, University of the Witwatersrand, Johannesburg, South Africa; j Department of Science/National Research Foundation: Vaccine Preventable Diseases, Faculty of Health Science, University of the Witwatersrand, Johannesburg, South Africa; k Pfizer Global Biometrics & Data Management, Pearl River, NY, USA; l Eagle Global Scientific, Atlanta, GA, USA; m Department of Clinical Microbiology, Christian Medical College, Vellore, India

**Keywords:** Group B streptococcus, maternal, neonatal, correlate of protection, vaccines

## Abstract

Measurement of IgG antibodies against group B streptococcus (GBS) capsular polysaccharide (CPS) by use of a standardized and internationally accepted multiplex immunoassay is important for the evaluation of candidate maternal GBS vaccines in order to compare results across studies. A standardized assay is also required if serocorrelates of protection against invasive GBS disease are to be established in infant sera for the six predominant GBS serotypes since it would permit the comparison of results across the six serotypes. We undertook an interlaboratory study across five laboratories that used standardized assay reagents and protocols with a panel of 44 human sera to measure IgG antibodies against GBS CPS serotypes Ia, Ib, II, III, IV, and V. The within-laboratory intermediate precision, which included factors like the lot of coated beads, laboratory analyst, and day, was generally below 20% relative standard deviation (RSD) for all six serotypes, across all five laboratories. The cross-laboratory reproducibility was < 25% RSD for all six serotypes, which demonstrated the consistency of results across the different laboratories. Additionally, anti-CPS IgG concentrations for the 44-member human serum panel were established. The results of this study showed assay robustness and that the resultant anti-CPS IgG concentrations were reproducible across laboratories for the six GBS CPS serotypes when the standardized assay was used.

## Introduction

Group B streptococcus (GBS) is a major cause of septicemia and meningitis in young infants, affecting 1–2 in every 1000 live births.^1^ It can present as early-  (< 7 days) or late-onset disease (7–89 days) in the first 3 months of life and is associated with adverse neurodevelopmental outcomes in up to 50% of meningitis survivors.^1^ It can also lead to intra-amniotic infection, bacteremia, and postpartum endometritis in pregnant women. The availability of a vaccine that could be given to pregnant women to provide direct protection for mothers and their infants through maternally transferred antibody is an urgent need. However, demonstrating efficacy for a GBS vaccine through disease endpoint efficacy trials would be logistically challenging, likely requiring enrollment of upwards of 80,000 participants, and several years to complete with significant operational challenges.^2^

An alternative approach, by identifying biomarkers that correlate with protection against disease, can facilitate the development and introduction of new vaccines via an immunological endpoint study, as is accepted for pneumococcal disease,^3^ *Haemophilus influenzae* type b disease,^4^ and meningococcal disease.^5–8^ An antibody threshold concentration for invasive pneumococcal disease based on serum anti-pneumococcal capsular polysaccharide (CPS) IgG was developed after protection had been demonstrated by the 7-valent^9,10^ and 9-valent^11^ conjugate vaccines in clinical efficacy studies. The aggregate IgG putative serocorrelate threshold was agreed by consensus to be 0·35 μg/mL based on results from three trials. Key to arriving at this estimate was a standardized ELISA that was adopted by the World Health Organization (WHO) for pneumococcal vaccine development in order to combine data from the different trials and laboratories.^12^ This internationally accepted standardized assay was transferred to multiple laboratories to facilitate measurement of IgG antibodies for serotype-specific pneumococcal CPS, and identification of a putative threshold of protection for pneumococcal disease.

For GBS, high concentrations of naturally occurring serotype-specific maternal antibody to CPS are associated with a reduced risk of invasive disease in neonates.^13^ Therefore, serum anti-CPS IgG concentration may be a relevant parameter to inform a reliable estimate of protection from invasive GBS disease. Over the last 40 years, 34 different quantitative assays have been described in the literature.^14^ Several studies have established an association between maternal or infant anti-CPS IgG concentrations and protection against GBS disease in infants, with some studies even reporting a protective threshold.^15–19^ However, the variability in assay methods and the lack of a human serum reference standard have precluded the definition of a globally accepted antibody threshold associated with risk reduction of invasive GBS disease. Thus, consensus is growing that standardization of assays to quantify GBS anti-CPS IgG responses to permit comparisons across natural history and vaccine studies would represent a major milestone for the definition of protective antibody concentrations that could be linked to vaccine performance.

A previously described multiplex immunoassay (MIA)^20^ was validated to measure IgG concentrations for the six predominant CPS serotypes of GBS (Ia, Ib, II, III, IV, and V), which are responsible for > 98% of GBS disease cases worldwide,^21^ and was adopted by the international GBS Consortium GASTON (Group B streptococcal Assay STandardisatiON)^22^ as the standardized assay. The assay will be used by members of GASTON to derive anti-CPS IgG concentrations from seroepidemiology studies and, consequently, allow comparison of results across studies and, importantly, laboratory settings. Additionally, the assay is being used to evaluate immune responses to an investigational GBS polysaccharide conjugate vaccine (termed GBS6)^19^, allowing the data from seroepidemiology studies generated to be directly compared to immunogenicity data from clinical vaccine trials.

To evaluate the reproducibility of the standardized assay across laboratories and to ensure that results between laboratories would be comparable, an interlaboratory study was undertaken. This report describes the concordance of results for the five laboratories that participated in the interlaboratory study of the GASTON assay.

## Materials and methods

### Study design

The GASTON assay, a validated six-plex anti-CPS IgG MIA, was transferred from Pfizer Inc. (Pearl River, New York, USA) to four study sites, including UK Health Security Agency (Porton Down, England), the Centers for Disease Control and Prevention (CDC) (Atlanta, Georgia, USA), St. George’s, University of London (London, England), and the Vaccines and Infectious Diseases Analytics Research Unit of the University of the Witwatersrand (Johannesburg, South Africa) during an 18-month period (07/2020 to 12/2021). All study sites completed and passed assay qualification testing prior to commencing the interlaboratory study. Verification that the assay was performing as expected post-assay transfer was conducted using a precision assessment with an 11-member human serum panel, composed of non-vaccinated human sera purchased from biorepositories and GBS6-vaccinated non-human primate (NHP) sera, and tested across 3 days with two laboratory analysts. For the interlaboratory study, participating sites conducted an analysis of a 44-member serum panel, comprised of 44 individual serum samples from GBS6-vaccinated, nonpregnant adult clinical study participants (NCT03170609), where all subjects underwent informed consent. The panel was tested in the assay 16 times by two analysts using two qualified bead lots to assess intra- and interlaboratory variability. The 44 serum samples were selected so that low, medium, and high concentration samples were present for each serotype. Bead lot qualification involved testing a previously qualified (reference) bead lot side-by-side with the new test lot of beads using a common serum panel in two independent assay runs. Upon comparing panel results for the assay using both the reference and test lot of beads, if the test lot of beads met the pre-defined acceptance criteria the new bead lot was determined to be qualified for use in the assay.

#### Ethical statements

All procedures performed on animals were in accordance with regulations and established guidelines and were reviewed and approved by the Institutional Animal Care and Use Committee (IACUC) or through an ethical review process.

#### Reagents

Participating sites were provided with critical reagents including quality control samples (QCS), which are pools of GBS6 immune human serum samples, uncoated MagPlex microspheres (beads), bovine serum albumin (BSA), a human serum reference standard, which is a pool of GBS6 immune human serum samples, secondary antibody, qualified beads and two serum panels that spanned the expected assay range, an 11-member and a 44-member serum panel as described above. Each laboratory site produced two lots of antigen-coated beads and qualified them against a previously qualified lot in a side-by-side comparison using the 11-member panel of GBS6-vaccinated NHP samples and non-vaccinated human serum samples. The 44-member serum panel was used to assess precision and reproducibility in the interlaboratory study. GBS CPS Poly-L-Lysine (CPS-PLL) conjugates used in the interlaboratory study were prepared for each serotype by MHRA and distributed to the participating laboratories for bead coating. A description of the bead coating process and GBS CPS-PLL preparation can be found in.^20^ The five participating laboratories supplied their own assay buffer and other materials necessary to perform the assay.

#### Standardised assay

The GASTON assay is an MIA based on Luminex MagPlex xMAP technology that allows for the measurement of GBS anti-CPS IgG (serotypes Ia, Ib, II, III, IV, and V) using six unique and spectrally distinct fluorescently dyed Luminex beads coupled to GBS CPS-PLL conjugates, as previously described.^20^ The standardized assay was run as previously described.^19,20^ Each 96-well assay plate included an 11-point human serum reference standard dilution series, quality control samples (QCS), and test serum samples. Representative reference standard serum dilution profiles for each of the six GBS CPS serotypes were previously described.^20^ Additionally, two wells containing assay buffer alone acted as blank controls. All samples and controls were diluted in an assay buffer (0.5% BSA in 10 mM PBS/0.05% Tween-20/0.02% NaN_3_, pH 7.2) on microtitter plates and incubated overnight. Test serum samples were tested in duplicate at the following dilutions: 1:500, 1:5,000, and 1:50,000. On day 2 of the assay, the plates were washed using a Tecan HydroSpeed™ plate washer (with magnetic bead attachment) to remove non-bound components. Following the wash step, a R-Phycoerythrin-conjugated goat anti-human IgG secondary antibody (Jackson ImmunoResearch, Cat. 109-115-098) was added to the plate. The plates were washed again as above and 100 µL/well was added after the last wash to resuspend the beads. The assay plates were read on a Luminex 200 reader using Bio-Plex Manager. The signal output was expressed as MFI, which was evaluated against the human serum reference standard curve with weight-based IgG assignments (in µg/mL) for each serotype, as previously described.^23^

#### Data and statistical analysis

Assay results were analyzed centrally using a validated SAS application to interpolate GBS anti-CPS IgG concentration data from the human serum reference standard using a log–log linear regression-based algorithm. Assay plates and samples which did not meet pre-defined system suitability criteria were considered invalid and were retested; system suitability criteria were based on reference curve parameters, internal QCS specification limits, and relevant aspects of the test samples. Intermediate precision was defined as the overall variability (days/operators, *etc*.) within the same laboratory (intra-laboratory precision).

To assess the intermediate precision within each of the test laboratories and within the reference laboratory, a variance component analysis (VCA) using a linear mixed model was used. For each laboratory, a model was fitted independently for each serotype and included analyst, day, and coated microsphere lot as random effects. This decomposition included the factors that related the reportable sample concentrations to the experimental design. The concentration data were analyzed after a log transformation, and the overall within laboratory percent relative standard deviation (%RSD) was determined by summing the variability (on the log scale) over each of the random effects and residual variability and back transforming this result^24^;  < 25% RSD was deemed acceptable as this is standard practice for binding assays.^24,25^

Additionally, antibody concentrations for the 44-member human serum interlaboratory study panel were estimated for the six CPS serotypes using the linear mixed model described above with the laboratory as an additional random effect included. The geometric mean concentration for each sample panel member, for each serotype, was calculated to generate consensus estimates and to evaluate agreement across laboratories. Consensus estimates of IgG concentrations of the 44-member human serum interlaboratory study panel were obtained by back transforming the estimated log-transformed concentration. For each of the 44 panel members, the %RSDs were also estimated.

Laboratory reproducibility was evaluated using the preceding linear mixed-effects model which included the laboratory as a random effect. The %RSD was determined by summing the variability over the random effects and back transforming the result.

To assess assay reproducibility between the five laboratories, variance decomposition of the total variability was performed for each serotype, separately. The decomposition included the factors that related the reportable sample concentrations to the experimental design for all laboratories. Additionally, agreement was assessed by the closeness of the (log) concentration between the two laboratories for each of the six serotypes as measured by the slope of the Deming regression line.^26^ For completeness, an analogous R^2^ formula is also provided in Appendix A. Scatter plots, including the Deming regression line, are provided to assess the ability of the five laboratories to produce consistent estimates of IgG concentrations of the 44-member human serum interlaboratory study panel for each serotype.

## Results

### Assay (intermediate) precision of laboratories (intra-laboratory precision)

Table A1 depicts the compiled results with intermediate precision from each laboratory. Intermediate precision was assessed by evaluating the amount of variability due to known sources: analysts, bead lot, and assay run date. The variability due to sources not included in the analysis was captured as residual variability. Across all laboratories and serotypes, assay run date and residual were the largest sources contributing to the total variability, while bead lot and analyst contributed least to the total variability. Our results indicated that each of the testing laboratories had comparable precision relative to the reference laboratory across all six serotypes, with no laboratory exceeding 21% RSD (Table A1). One laboratory (Lab #2) did not complete the full experimental design due to disruptions caused by the COVID-19 pandemic and thus had approximately one-third of the maximum number of reportable sample concentrations.

### Correlation between antibody concentrations across laboratories, by serotype

**Figure 1.**
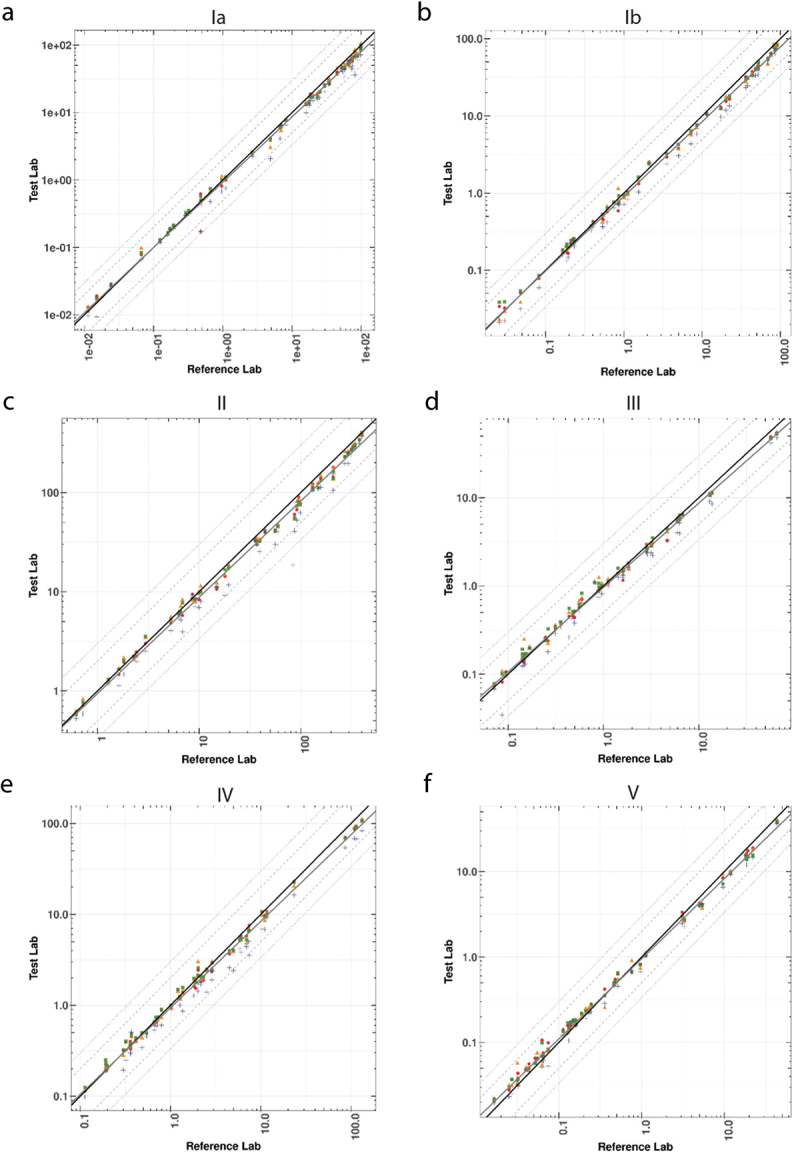
Deming regression of GBS Ia, Ib, II, III, IV, and V between four laboratories and the reference laboratory. A scatter plot for sample member pairs between all test labs (y-axis) and the reference laboratory (x-axis) is shown for GBS CPS serotypes Ia (a), Ib (b), II (c), III (d), IV (e), and V (f). Each test laboratory is represented by a symbol: lab 1 (red circle), lab 2 (orange triangle), lab 3 (green square), and lab 4 (blue plus sign). The solid black diagonal line provides a line of perfect agreement (slope = 1, intercept = 0) with the Deming regression line (solid gray line). Lines for two and three-fold differences are also shown as the dashed and dotted lines, respectively, for reference.

The Deming regression plots (Figure 1) demonstrate a strong correlation between all test laboratories that participated in the interlaboratory study and the reference laboratory. These plots illustrate the level of agreement between the test laboratories and the reference laboratory for serotype Ia, Ib, II, III, IV, and V. Divergence in IgG concentrations was more often observed at the upper end of the assay range and may have been due to laboratory-specific conditions. Table 1 summarizes the Deming regression statistics and analogous R^2^ for each serotype from all laboratories combined.

**Table 1.** Deming regression statistics and analogous R^2^ per serotype for all laboratories combined.

GBS Serotype	Slope^a^	Intercept^a^	Analogous R^2 b^
Ia	0.9685	−0.0321	0.9988
Ib	0.968	−0.0429	0.999
II	0.9701	−0.0267	0.9976
III	0.956	−0.0177	0.9977
IV	0.9519	−0.0279	0.9976
V	0.9384	−0.0209	0.999

^a^The slope and intercept were calculated using Deming Regression.

^b^Analogous R^2^ formula can be found in Appendix A.

### Laboratory reproducibility

Interlaboratory precision or reproducibility of the standardized GASTON assay was assessed. Assay reproducibility across all six serotypes was strong, with no serotype exceeding 24% total %RSD (Table 2 and Table A2 for the consensus serotype-specific IgG estimates for the 44-member human serum interlaboratory study panel; data presented are a breakdown of the variability presented in Table 2). By combining the data across all laboratories and structuring the analysis to identify the factors that contribute to the assay variability (%RSD), throughout the study duration, aside from residual, the laboratory contributed the most to assay variability, followed by analyst then bead lot for all serotypes (Table 2). The reportable sample concentrations for the 44-member panel from Laboratory #4 were consistently lower than those from the other four laboratories. Therefore, to prevent biasing the consensus estimates low, all data from Laboratory #4 were excluded from the consensus estimates (Table A2). A sensitivity analysis determined that depending on the serotype, estimates would be 2.3–5.6% lower across serotypes if Laboratory #4 results were included. After investigation, no conclusive evidence identified the root cause for lower sample concentrations from this laboratory. Although the age of the beads used by this laboratory was greater compared to other laboratories, this should not have given rise to appreciably different assay results upon normalization to the standard curve. The overall variability was shown to be comparable to what has previously been accepted for a Luminex-based immunoassay platform.^25^

**Table 2.** Reproducibility across laboratories and serotype as %RSD.

GBS Serotype	N^a^	%RSD					
		Day^b^	Laboratory^b^	Analyst^b^	Bead Lot^b^	Residual^c^	Total^d^
Ia	3182	0.0	10.1	7.7	5.1	19.2	23.7
Ib	3173	4.9	10.4	5.7	3.4	14.0	19.4
II	3129	0.0	11.0	7.1	4.6	12.8	19.0
III	3064	6.2	8.9	7.6	2.8	15.6	20.8
IV	3329	4.1	12.6	5.8	5.2	15.0	21.7
V	3172	3.9	5.6	7.0	4.1	15.2	18.6

^a^N represents the total number of sample determinations across all laboratories, per GBS serotype.

^b^‘Day,’ ‘Laboratory,’ ‘Analyst’ and ‘Bead lot’ are factors that may contribute to the assay variability throughout the study duration.

^c^‘Residual’ represents variability not accounted for by the factors specified.

^d^‘Total’ can be defined as the total variability from all the components included in the variance decomposition analysis.

## Discussion

To our knowledge, this is the first time that such a consortium has come together to test standardized reagents and protocols for the assessment of IgG antibodies against GBS.

Use of a standardized assay across the worldwide GBS research community will permit comparison of assay results from multiple laboratories that test human serum samples, for example, from seroepidemiology studies that are being conducted across populations with different ethnicities and geographical locations. Such a consensus is key to identifying a globally accepted antibody threshold associated with risk reduction of invasive GBS disease that has not previously been possible. A harmonized assay to evaluate sera from seroepidemiology studies around the world will allow for a better understanding of how natural immunity relates to the immune response elicited by GBS polysaccharide conjugate vaccines. Defining an internationally accepted serological protective anti-CPS antibody concentration for invasive GBS disease in early infancy using a standardized assay may be applied to vaccine development and could be used to define an immunological endpoint in Phase 3 studies, possibly avoiding the need for a large field efficacy trial with disease endpoints.

A validated, standardized Luminex-based immunoassay that was developed to quantify serotype-specific anti-CPS GBS IgG^19,20,27^ was transferred to four laboratories across three continents. Assessment of inter- and intra-laboratory precision results indicated excellent reproducibility, intermediate precision, and ultimately assay transferability and portability. Intra-laboratory intermediate precision was comparable across the participating laboratories and inter-laboratory reproducibility showed a high degree of agreement with consensus IgG concentration estimates. To further underscore the robustness of the assay, a 4-parameter logistic regression curve fit algorithm was used to calculate antigen-specific IgG concentrations from mean fluorescence intensity (MFI) data generated from the interlaboratory study as an exploratory study objective. By using the same system suitability criteria generated from the assay validation,^20^ the key study outcomes, including comparable inter- and intra-laboratory precision, remained consistent and demonstrated assay robustness. Having a common standardized assay has proven to be an effective tool for the establishment of a protective antibody threshold for pneumococcal disease^12^ and now GBS has the potential to follow the same path.

Successful completion of the interlaboratory study and demonstration that the assay was robust and transferable across laboratories, was the culmination of many necessary steps required by each of the laboratories, including, but not limited to, coordination of assay-specific training, reagent transfer, and microsphere preparation and qualification. It is recommended that any future laboratory that enlists the standardized assay for research and/or clinical testing purposes should qualify and evaluate the precision and reproducibility of the assay, as described here. This would ensure that the standardized assay is performed with the highest degree of quality, and furthermore, ensure that results generated can be interpreted appropriately and compared across laboratories.

Current efforts are underway to develop a large-volume international human serum reference standard with weight-based serotype-specific IgG assignments for use by the GBS research and scientific community. Calibration of the international reference standard is planned to be performed using a modern approach for quantifying serotype-specific IgG in human serum that would allow for comparison of IgG concentrations across serotypes.^23^ The successes brought to assay standardization for pneumococcal vaccines by the human serum reference standards 89SF and 007sp highlight the urgent need for this critical reagent for GBS. An international human serum reference standard for GBS could promote global comparisons of serotype-specific anti-CPS IgG within and between assay platforms and consequently strengthen the ability to interpret clinical and seroepidemiology study results across laboratory sites worldwide.

Another much needed tool that is currently under development in order to monitor the standardized assay long-term is creation of a large volume human serum panel that could be used to assess assay performance on an annual basis. The goal of creating this human serum panel is similar in nature to the WHO calibration sera used during the immunological bridging of pneumococcal reference standard serum 89SF to 007sp in that IgG results from each individual panel member, and combined all together, could be compared across laboratories to serve as a measure of assay robustness. For example, this human serum proficiency panel would be provided to all laboratories that successfully demonstrate precision and reproducibility of the standardized assay and would ensure that the assay is performing as expected over time, regardless of laboratory analyst, microsphere preparation, or reagent lot changes. A future study is planned by the GBS Consortium to assign serotype-specific IgG concentration to this panel to assess and monitor changes in assay performance over time.

While the interlaboratory study described here was a success and demonstrated that the assay was precise and reproducible within and across laboratories, there were certain limiting aspects of the study, which we describe here. Each laboratory was requested to complete the interlaboratory study within 8 weeks from the date of final bead pooling. Three out of the five participating laboratories completed the study in this time frame, which would indicate that the study was performed with beads of similar age at each of these laboratories. One laboratory was not able to complete the study within the requested time window (Laboratory #4) and one laboratory (Laboratory #2) did not complete the full experimental design and thus had approximately one-third of the maximum number of reportable sample concentrations. Laboratory #4 experienced numerous COVID-19 induced shipping delays and site closures between July 2020 and December 2021 that caused the study to be paused and restarted on multiple occasions. Consequently, this laboratory performed the study with beads that were outside the predefined criteria pertaining to the age of the beads. Additional considerations of the GASTON assay include the requirement for other potential global users of the assay to formally join the GBS Consortium, and subsequent completion of training and implementation. In addition, further work is ongoing to make a data analysis template freely available for laboratories that are unable to access the SAS program, as well as development of the international reference standard, currently underway within GASTON.

The standardized, multiplex GASTON immunoassay showed strong concordance between sites across the assay range and serotypes and was thus determined to be robust and transferable across the different laboratories. The assay will be used in the future to determine serocorrelates of protection against invasive infant GBS disease in natural immune sera. This assay will also make it possible to link such data to other data generated by the same assay, including studies evaluating immune responses to GBS CPS conjugate vaccines in clinical development.

## Supplementary Material

Supplemental Material
