# Supplementary material for: Interlaboratory comparison of a multiplex immunoassay that measures human serum IgG antibodies against six-group B streptococcus polysaccharides
Source: Hum Vaccin Immunother. 2024 Apr 12;20(1):2330138. doi: 10.1080/21645515.2024.2330138 (PMC11018077; doi:10.1080/21645515.2024.2330138)
Supplement: Supplemental Material [file KHVI_A_2330138_SM6094.pdf]

SUPPLEMENTARY MATERIALS

Appendix A

1. Analogous R^2 formula

The analogous R^2 statistic is defined as:

$$R^2 = \frac{\lambda\sum(\hat{X}_i - \bar{X})^2 + \sum(\hat{Y}_i - \bar{Y})^2}{\lambda\sum(X_i - \bar{X})^2 + \sum(Y_i - \bar{Y})^2}$$

where:

$\hat{Y}_i$ = Fitted values for Y

$\hat{X}_i$ = Fitted values for X

λ= ratio of variance of Y to the variance of X

The analogous R^2 statistic has the usual R^2 interpretation but accounts for errors in both X and Y simultaneously.

Information Classification: General

Appendix B

Table A1. Assay (intermediate) precision of each laboratory.

Source[b]	GBS Serotype[c]	Covariance Parameter[d]	Variance[e]	Standard Deviation[f]	Percent of Total Variability[g]	%RSD[h]
Laboratory 1	Ia	Analyst	0.0000	0.0000	0.000	0.0
		Bead Lot	0.0000	0.0000	0.000	0.0
		Date (Analyst)[a]	0.0021	0.0462	47.610	10.7
		Residual	0.0023	0.0485	52.390	11.2
		Total	0.0045	0.0670	100.000	15.5
Laboratory 2	Ia	Analyst	0.0000	0.0000	0.000	0.0
		Bead Lot	0.0000	0.0000	0.000	0.0
		Date (Analyst)	0.0006	0.0238	15.040	5.5
		Residual	0.0032	0.0566	84.960	13.1
		Total	0.0038	0.0614	100.000	14.2
Laboratory 3	Ia	Analyst	0.0000	0.0000	0.000	0.0
		Bead Lot	0.0002	0.0142	2.651	3.3
		Date (Analyst)	0.0006	0.0247	7.983	5.7
		Residual	0.0068	0.0825	89.366	19.2
		Total	0.0076	0.0873	100.000	20.3
Laboratory 4	Ia	Analyst	0.0000	0.0000	0.000	0.0

Information Classification: General

Source[b]	GBS Serotype[c]	Covariance Parameter[d]	Variance[e]	Standard Deviation[f]	Percent of Total Variability[g]	%RSD[h]
		Bead Lot	0.0000	0.0024	0.075	0.5
		Date (Analyst)	0.0014	0.0369	18.427	8.5
		Residual	0.0060	0.0777	81.497	18.0
		Total	0.0074	0.0861	100.000	20.0
Reference Laboratory	Ia	Analyst	0.0000	0.0000	0.000	0.0
		Bead Lot	0.0009	0.0304	19.466	7.0
		Date (Analyst)	0.0015	0.0388	31.748	8.9
		Residual	0.0023	0.0481	48.785	11.1
		Total	0.0047	0.0688	100.000	15.9
Laboratory 1	Ib	Analyst	0.0001	0.0074	1.485	1.7
		Bead Lot	0.0000	0.0000	0.000	0.0
		Date (Analyst)	0.0020	0.0451	55.654	10.4
		Residual	0.0016	0.0396	42.861	9.1
		Total	0.0037	0.0605	100.000	14.0
Laboratory 2	Ib	Analyst	0.0000	0.0000	0.000	0.0
		Bead Lot	0.0000	0.0000	0.000	0.0
		Date (Analyst)	0.0005	0.0229	18.971	5.3
		Residual	0.0022	0.0473	81.029	10.9

Information Classification: General

Source[b]	GBS Serotype[c]	Covariance Parameter[d]	Variance[e]	Standard Deviation[f]	Percent of Total Variability[g]	%RSD[h]
		Total	0.0028	0.0526	100.000	12.1
Laboratory 3	Ib	Analyst	0.0000	0.0000	0.000	0.0
		Bead Lot	0.0000	0.0060	1.335	1.4
		Date (Analyst)	0.0006	0.0252	23.766	5.8
		Residual	0.0020	0.0447	74.898	10.3
		Total	0.0027	0.0516	100.000	11.9
Laboratory 4	Ib	Analyst	0.0000	0.0000	0.000	0.0
		Bead Lot	0.0000	0.0040	0.358	0.9
		Date (Analyst)	0.0010	0.0309	21.920	7.1
		Residual	0.0034	0.0582	77.722	13.5
		Total	0.0044	0.0660	100.000	15.3
Reference Laboratory	Ib	Analyst	0.0000	0.0000	0.000	0.0
		Bead Lot	0.0004	0.0196	8.830	4.5
		Date (Analyst)	0.0016	0.0403	37.310	9.3
		Residual	0.0023	0.0484	53.860	11.2
		Total	0.0044	0.0660	100.000	15.3
Laboratory 1	II	Analyst	0.0000	0.0000	0.000	0.0
		Bead Lot	0.0000	0.0000	0.000	0.0

Information Classification: General

Source[b]	GBS Serotype[c]	Covariance Parameter[d]	Variance[e]	Standard Deviation[f]	Percent of Total Variability[g]	%RSD[h]
		Date (Analyst)	0.0023	0.0475	58.366	11.0
		Residual	0.0016	0.0401	41.634	9.3
		Total	0.0039	0.0622	100.000	14.4
Laboratory 2	II	Analyst	0.0000	0.0000	0.000	0.0
		Bead Lot	0.0000	0.0000	0.000	0.0
		Date (Analyst)	0.0016	0.0397	33.964	9.2
		Residual	0.0031	0.0554	66.036	12.8
		Total	0.0046	0.0681	100.000	15.8
Laboratory 3	II	Analyst	0.0001	0.0104	5.422	2.4
		Bead Lot	0.0000	0.0000	0.000	0.0
		Date (Analyst)	0.0005	0.0221	24.450	5.1
		Residual	0.0014	0.0375	70.128	8.6
		Total	0.0020	0.0447	100.000	10.3
Laboratory 4	II	Analyst	0.0000	0.0000	0.000	0.0
		Bead Lot	0.0001	0.0122	4.622	2.8
		Date (Analyst)	0.0011	0.0330	33.732	7.6
		Residual	0.0020	0.0446	61.646	10.3
		Total	0.0032	0.0568	100.000	13.1

Information Classification: General

Source[b]	GBS Serotype[c]	Covariance Parameter[d]	Variance[e]	Standard Deviation[f]	Percent of Total Variability[g]	%RSD[h]
Reference Laboratory	II	Analyst	0.0000	0.0000	0.000	0.0
		Bead Lot	0.0001	0.0086	2.305	2.0
		Date (Analyst)	0.0017	0.0407	51.537	9.4
		Residual	0.0015	0.0385	46.158	8.9
		Total	0.0032	0.0566	100.000	13.1
Laboratory 1	III	Analyst	0.0001	0.0093	1.899	2.1
		Bead Lot	0.0000	0.0000	0.000	0.0
		Date (Analyst)	0.0017	0.0408	36.315	9.4
		Residual	0.0028	0.0532	61.787	12.3
		Total	0.0046	0.0677	100.000	15.7
Laboratory 2	III	Analyst	0.0000	0.0000	0.000	0.0
		Bead Lot	0.0000	0.0000	0.000	0.0
		Date (Analyst)	0.0026	0.0512	40.835	11.8
		Residual	0.0038	0.0616	59.165	14.3
		Total	0.0064	0.0801	100.000	18.6
Laboratory 3	III	Analyst	0.0000	0.0000	0.000	0.0
		Bead Lot	0.0004	0.0188	10.974	4.3
		Date (Analyst)	0.0009	0.0297	27.550	6.9

Information Classification: General

Source[b]	GBS Serotype[c]	Covariance Parameter[d]	Variance[e]	Standard Deviation[f]	Percent of Total Variability[g]	%RSD[h]
		Residual	0.0020	0.0444	61.476	10.3
		Total	0.0032	0.0567	100.000	13.1
Laboratory 4	III	Analyst	0.0000	0.0000	0.000	0.0
		Bead Lot	0.0000	0.0000	0.000	0.0
		Date (Analyst)	0.0018	0.0423	36.438	9.8
		Residual	0.0031	0.0559	63.562	12.9
		Total	0.0049	0.0701	100.000	16.2
Reference Laboratory	III	Analyst	0.0000	0.0000	0.000	0.0
		Bead Lot	0.0014	0.0368	20.041	8.5
		Date (Analyst)	0.0020	0.0448	29.747	10.3
		Residual	0.0034	0.0582	50.212	13.5
		Total	0.0067	0.0821	100.000	19.1
Laboratory 1	IV	Analyst	0.0000	0.0042	0.480	1.0
		Bead Lot	0.0001	0.0109	3.256	2.5
		Date (Analyst)	0.0018	0.0429	50.952	9.9
		Residual	0.0016	0.0405	45.312	9.3
		Total	0.0036	0.0601	100.000	13.9
Laboratory 2	IV	Analyst	0.0000	0.0000	0.000	0.0

Information Classification: General

Source[b]	GBS Serotype[c]	Covariance Parameter[d]	Variance[e]	Standard Deviation[f]	Percent of Total Variability[g]	%RSD[h]
		Bead Lot	0.0000	0.0000	0.000	0.0
		Date (Analyst)	0.0006	0.0237	18.284	5.5
		Residual	0.0025	0.0502	81.716	11.6
		Total	0.0031	0.0555	100.000	12.8
Laboratory 3	IV	Analyst	0.0000	0.0000	0.000	0.0
		Bead Lot	0.0000	0.0000	0.000	0.0
		Date (Analyst)	0.0005	0.0224	15.514	5.2
		Residual	0.0027	0.0523	84.486	12.1
		Total	0.0032	0.0569	100.000	13.2
Laboratory 4	IV	Analyst	0.0000	0.0000	0.000	0.0
		Bead Lot	0.0009	0.0305	16.702	7.0
		Date (Analyst)	0.0017	0.0408	29.925	9.4
		Residual	0.0030	0.0545	53.373	12.6
		Total	0.0056	0.0746	100.000	17.3
Reference Laboratory	IV	Analyst	0.0000	0.0000	0.000	0.0
		Bead Lot	0.0009	0.0304	13.621	7.0
		Date (Analyst)	0.0017	0.0409	24.765	9.4
		Residual	0.0042	0.0646	61.614	15.0

Information Classification: General

Source[b]	GBS Serotype[c]	Covariance Parameter[d]	Variance[e]	Standard Deviation[f]	Percent of Total Variability[g]	%RSD[h]
		Total	0.0068	0.0823	100.000	19.1
Laboratory 1	V	Analyst	0.0000	0.0000	0.000	0.0
		Bead Lot	0.0000	0.0000	0.000	0.0
		Date (Analyst)	0.0049	0.0703	64.609	16.3
		Residual	0.0027	0.0520	35.391	12.0
		Total	0.0077	0.0875	100.000	20.3
Laboratory 2	V	Analyst	0.0000	0.0000	0.000	0.0
		Bead Lot	0.0003	0.0171	5.505	3.9
		Date (Analyst)	0.0015	0.0386	28.083	8.9
		Residual	0.0035	0.0593	66.412	13.7
		Total	0.0053	0.0728	100.000	16.9
Laboratory 3	V	Analyst	0.0000	0.0000	0.000	0.0
		Bead Lot	0.0000	0.0000	0.000	0.0
		Date (Analyst)	0.0005	0.0231	26.444	5.3
		Residual	0.0015	0.0385	73.556	8.9
		Total	0.0020	0.0449	100.000	10.4
Laboratory 4	V	Analyst	0.0000	0.0000	0.000	0.0
		Bead Lot	0.0000	0.0062	0.858	1.4
		Date (Analyst)	0.0009	0.0306	21.187	7.1

Information Classification: General

Source[b]	GBS Serotype[c]	Covariance Parameter[d]	Variance[e]	Standard Deviation[f]	Percent of Total Variability[g]	%RSD[h]
		Residual	0.0035	0.0587	77.956	13.6
		Total	0.0044	0.0665	100.000	15.4
Reference Laboratory	V	Analyst	0.0000	0.0000	0.000	0.0
		Bead Lot	0.0005	0.0224	9.953	5.2
		Date (Analyst)	0.0022	0.0466	43.125	10.8
		Residual	0.0024	0.0486	46.922	11.2
		Total	0.0050	0.0710	100.000	16.5

[a]'Date (Analyst)' parameter indicates 'days' are represented in the analysis design as unique to the 'analyst'. The %RSD associated with 'Date (Analyst)' is the total variability due to day-to-day effects across all analysts. [b]The Source column represents the laboratory that performed the experimental runs.

[c]The GBS Serotype column represents the GBS serotype the data pertains to.

[d]The Covariance Parameter represents the source of variation for the assay, for example, the role a laboratory analyst may have in assay variability.

[e]The Variance column represents the distance between each number in a dataset from its mean.

[f]Standard deviation is the square root of the variance. Variance and standard deviation are based on log transformed data.

Information Classification: General

[g]The Percent of Total Variability represents the percent variability that each covariance parameter (laboratory analyst, bead lot, date or residual) contributes to the total.

[h]Additional details for the %RSD calculation can be found in [24].

Information Classification: General

Table A2. Consensus IgG assignments for the interlaboratory study precision panel.

Sample	GBS Ia			GBS Ib			GBS II			GBS III			GBS IV			GBS V		
	N[a]	GM[b]	%RSD[c]	N	GM	%RSD	N	GM	%RSD	N	GM	%RSD	N	GM	%RSD	N	GM	%RSD
1	56	29.982	16.9	58	0.184	16.3	57	32.979	13.6	59	0.483	16.4	59	1.423	13.4	57	0.139	18.3
2	61	0.079	25.7	60	1.472	18.2	60	15.956	19.3	62	0.154	25.7	62	0.310	13.8	61	0.357	21.9
3	48	0.012	21.0	55	7.772	10.9	55	3.197	12.4	6	1.009	14.1	56	0.362	15.9	56	0.082	37.3
4	57	48.410	11.6	56	0.502	13.2	55	182.655	12.7	57	57.162	12.4	57	114.908	12.0	54	0.028	22.7
5	52	0.017	68.0	57	17.064	15.7	56	6.437	13.7	32	4.070	34.9	57	2.445	13.3	58	0.146	23.2
6	56	1.062	16.3	19	87.621	10.9	56	34.020	12.4	54	0.090	22.0	58	0.480	12.7	1	45.241	NC[d]
7	39	102.080	10.1	58	4.201	18.6	58	143.243	13.2	59	1.453	14.5	59	4.005	15.3	60	0.020	17.0
8	58	62.912	16.8	55	65.548	13.1	57	2.186	11.0	58	0.478	12.1	59	0.507	10.1	58	0.226	17.2
9	56	58.475	14.7	58	6.245	17.8	56	8.510	16.3	59	2.687	17.7	58	5.729	19.2	58	16.606	21.7
10	58	6.339	14.3	54	56.702	17.4	57	0.590	10.6	58	0.991	11.5	59	9.751	14.4	57	4.344	18.9
11	54	19.341	18.3	57	0.170	19.3	57	10.181	18.5	58	0.971	21.4	58	10.746	18.7	56	18.329	20.5
12	59	21.478	14.3	56	43.787	14.9	57	313.796	10.1	59	11.947	15.8	60	0.208	30.0	60	2.838	18.7
13	56	30.360	14.2	59	0.929	14.9	57	118.181	14.4	59	5.979	11.5	59	2.403	26.2	57	4.326	14.2
14	57	0.026	17.1	54	76.487	11.2	57	6.815	30.7	60	0.701	26.5	59	6.951	13.5	58	0.130	12.8
15	54	0.677	27.2	58	32.421	15.1	56	11.937	19.1	58	2.939	11.9	57	1.748	13.8	56	0.714	14.9
16	58	48.097	17.1	55	0.367	11.3	56	82.420	16.5	57	11.365	14.6	59	0.414	12.7	58	0.166	11.5
17	59	0.944	20.4	33	84.992	9.5	57	260.977	13.7	59	0.476	25.4	60	92.933	16.1	59	0.235	11.5
18	56	65.651	16.3	56	0.032	18.5	51	358.321	10.1	59	0.261	30.3	60	1.794	14.7	57	0.058	15.1
19	55	18.060	15.0	58	2.329	15.2	57	1.936	14.6	58	0.189	13.2	58	1.383	15.6	54	0.046	14.1
20	54	0.308	12.0	58	0.748	18.7	56	6.037	12.0	57	0.248	10.7	57	5.645	15.7	35	38.400	9.7

Information Classification: General

Sample	GBS Ia			GBS Ib			GBS II			GBS III			GBS IV			GBS V		
	N[a]	GM[b]	%RSD[c]	N	GM	%RSD	N	GM	%RSD	N	GM	%RSD	N	GM	%RSD	N	GM	%RSD
21	56	57.038	12.9	58	0.488	15.9	58	119.265	14.3	60	2.728	22.1	60	0.434	46.4	58	0.154	15.3
22	59	4.100	26.3	55	13.705	24.1	58	126.690	21.5	59	50.144	22.2	60	7.147	22.7	57	0.037	22.2
23	55	0.182	10.7	57	0.222	10.8	57	0.734	16.6	58	0.152	15.8	57	0.205	9.3	56	10.234	13.5
24	57	50.547	12.5	56	42.278	12.0	55	245.209	11.2	58	0.587	16.5	57	10.395	13.6	56	0.035	22.5
25	58	48.363	13.7	59	0.758	26.7	57	41.731	12.5	58	6.545	12.1	58	2.934	13.3	59	16.108	13.6
26	58	14.041	15.6	58	31.689	15.9	58	1.596	12.1	58	0.074	12.4	59	0.840	12.3	59	0.084	23.0
27	57	71.091	12.2	56	46.061	12.1	55	300.734	10.1	56	2.693	16.3	57	22.529	13.0	56	0.063	16.1
28	58	18.132	14.2	58	10.847	12.9	57	64.122	24.6	58	3.240	19.8	59	0.976	12.8	58	0.514	13.4
29	60	15.054	18.0	56	47.673	14.1	25	413.073	7.0	22	79.306	11.3	61	0.634	14.6	59	15.891	17.5
30-	59	26.333	13.9	59	0.238	12.8	58	259.420	13.9	59	1.380	18.7	60	0.713	11.3	61	0.268	12.6
31	57	7.812	9.9	58	0.834	14.3	58	1.284	10.6	59	0.100	11.5	59	0.693	11.4	60	0.161	14.0
32	57	6.188	14.4	56	0.609	10.2	55	76.976	16.1	57	5.645	13.9	57	0.116	9.8	57	0.846	13.6
33	60	0.160	15.1	54	0.239	14.7	59	9.253	18.0	60	0.161	13.6	61	5.362	16.1	57	0.049	19.5
34	50	77.897	15.0	55	0.082	20.6	1	66.288	NC[d]	58	0.157	20.7	57	73.835	12.8	58	0.033	19.5
35	55	2.522	12.0	54	0.049	17.9	54	87.009	12.4	55	0.150	13.3	56	4.245	15.7	56	3.069	14.5
36	59	0.493	9.8	57	1.027	11.6	58	33.785	9.7	58	0.930	14.6	59	0.378	10.4	59	0.065	12.3
37	59	0.479	8.4	58	37.540	14.4	57	282.005	11.6	58	1.730	12.8	59	2.057	13.6	58	0.479	10.5
38	56	0.124	10.9	55	0.188	13.6	55	44.720	17.4	56	1.543	10.3	56	0.347	10.1	56	0.172	9.3
39	59	0.476	49.8	57	0.031	24.8	57	49.823	15.5	3	8.066	5.8	59	2.096	10.8	57	0.591	18.0
40	59	0.206	11.8	59	0.413	11.0	57	18.141	14.3	58	0.161	13.3	58	0.214	13.3	59	0.208	12.0

Information Classification: General

Sample	GBS Ia			GBS Ib			GBS II			GBS III			GBS IV			GBS V		
	N[a]	GM[b]	%RSD[c]	N	GM	%RSD	N	GM	%RSD	N	GM	%RSD	N	GM	%RSD	N	GM	%RSD
41	51	92.701	12.4	45	79.760	9.0	57	155.778	21.8	59	6.002	18.0	60	1.986	11.3	58	0.167	13.3
42	55	40.683	13.3	54	3.184	13.1	54	5.126	12.6	56	1.110	10.8	55	2.531	15.1	55	8.421	20.4
43	59	0.336	12.9	58	0.197	11.2	57	8.735	10.4	59	0.377	10.8	59	1.226	10.0	59	0.070	15.1
44	55	74.924	13.6	54	18.267	18.4	59	2.356	11.5	60	0.329	18.4	59	96.948	14.5	60	1.058	14.5

[a]N represents the number of determinations across all laboratories, per sample.

[b]GM represents the geometric mean IgG concentration for the respective sample and serotype.

[b]Additional details for the %RSD calculation can be found in [24].

[d]NC, not calculable.

Information Classification: General
